# Type D personality to insomnia: Sleep reactivity, sleep effort, and sleep hygiene as mediators

**DOI:** 10.3389/fpsyt.2023.1160772

**Published:** 2023-04-11

**Authors:** Omer Faruk Uygur, Oli Ahmed, Hilal Uygur, Aynur Bahar, Onur Hursitoglu, Seockhoon Chung, Christopher L. Drake

**Affiliations:** ^1^Department of Psychiatry, Ataturk University School of Medicine, Erzurum, Türkiye; ^2^Department of Psychology, University of Chittagong, Chattogram, Bangladesh; ^3^Department of Psychiatry, Erzurum Training and Research Hospital, Erzurum, Türkiye; ^4^Department of Psychiatric Nursing, Gaziantep University Faculty of Health Sciences, Gaziantep, Türkiye; ^5^Department of Psychiatry, Sular Academy Hospital, Kahramanmaras, Türkiye; ^6^Department of Psychiatry, Asan Medical Center, University of Ulsan College of Medicine, Seoul, Republic of Korea; ^7^Henry Ford Hospital Sleep Disorders and Research Center, Detroit, MI, United States; ^8^Department of Psychiatry and Behavioral Neurosciences, Wayne State College of Medicine, Detroit, MI, United States

**Keywords:** Type D personality, insomnia, sleep reactivity, sleep effort, sleep hygiene

## Abstract

**Background:**

Insomniacs are heterogenous group with very diverse personalities. We aimed to investigate the mediating role of sleep reactivity (SR), sleep hygiene (SH), and sleep effort (SE) in the relationship between Type D personality and insomnia.

**Materials and methods:**

We conducted a cross-sectional survey among 474 participants. The survey comprised the sociodemographic data form, Insomnia Severity Index (ISI), D Type Personality Scale (DS-14), Ford Insomnia Response to Stress Test (FIRST), Glasgow Sleep Effort Scale (GSES), and Sleep Hygiene Index (SHI). We conducted hierarchical multiple regression analysis to identify the associations between age, sex, SR, Type D personality traits, SE, SH, and insomnia severity. We subsequently conducted mediation analyses to examine whether SR, SH, and SE mediated the relationship between Type D personality and insomnia.

**Results:**

ISI, DS-14, FIRST, SHI, and GSES scores were significantly higher in individuals with Type D personality. Female sex, SR, Type D personality traits, SE, and SH explained 45% of the variance in insomnia severity. When age, sex, insomnia response to stress, and Type D personality traits were controlled, SE and SH significantly explained 25% of the variance in insomnia severity (*R*^2^ = 0.45, *R*^2^ change = 0.25, *F* (6.474) = 65.58, *p* < 0.001). SR, SE, and SH each played a partial mediating role between Type D personality and insomnia.

**Conclusion:**

The findings showed that individuals with Type D personality had high SR and that individuals with a higher number of these personality traits exhibited more severe insomnia symptoms through high SR, greater SE, and worse SH.

## Introduction

Insomnia is a common problem worldwide as a symptom or disorder, with a prevalence of 30 and 10%, respectively (1, 2). It is considered a common symptom of nearly all mental health disorders (3). Insomnia disorder is characterized by difficulty falling and/or staying asleep; it is further characterized by awakening earlier in the morning, with the inability to fall back to sleep at least 3 nights per week and for at least 3 months (4). Investigating this disorder is highly important for public health, since it is associated with more hospital visits and loss of workforce due to daytime sleepiness, fatigue, impairment in cognitive performance, and mood disturbances (3, 5–7).

Personality traits may be a predisposing and potentially perpetuating factor for insomnia (8). Type D personality, also called “distressed” personality, is the tendency to experience negative emotions and avoid social interactions for fear of rejection or disapproval by others (9). This personality is defined by the synergistic combination of two stable traits, negative affectivity (NA) and social inhibition (SI) (9). NA means experiencing negative emotions such as sadness, anger, and worry. SI is defined as the inhibition of emotions and behavioral expressions in social settings (9). Many studies have shown that the Type D construct is a predictor of psychological distress and morbidity in cardiac patients (10). Additionally, regarding the general population, it has been concluded that the Type D construct can be used as a predictor of depression, anxiety, and social anxiety (11, 12). Neuroticism has been considered a fundamental personality trait among the Big Five dimensions (13). Individuals with high scores for neuroticism are more likely than average to be moody and experience feelings such as anxiety, worry, fear, anger, frustration, envy, jealousy, guilt, depressed mood, and loneliness. In general, people with insomnia show signs of high neuroticism (14). For example, in a sample of 77 undergraduates, Williams and Moroz found that neuroticism was positively associated with sleep dysfunction (15). Type D personality traits overlap with neuroticism and extraversion. People with Type D personality score high on neuroticism, which is reflected in NA; they score low on extraversion, which is reflected in SI. This further suggests that the Type D personality can be represented by the combination of low extraversion and high neuroticism (16). Various studies have shown that insomnia is related to NA, SI, internalization, and anxious concerns. Indeed, a study has shown that Type D personality is a predictor for sleep disturbances in the police and nurse population (17). Another study found that adolescents with Type D personality had a fourfold increased risk of experiencing insomnia (17). In a recent study, the severity of insomnia was found to be significantly higher in those with Type D personality than in those without (18).

The major predictive factor for insomnia is sleep reactivity (SR) (19). SR should be evaluated as the possibility of experiencing insomnia during stressful life events. Individuals with high sleep reactivity are prone to develop insomnia (19). Sleep reactivity is evaluated with the 9-item FIRST scale developed by Drake et al. (20). It has been shown that sleep reactivity is genetic, insomnia persists in individuals with high sleep reactivity despite the disappearance of stress, and that these individuals are resistant to cognitive behavioral therapy for insomnia (21). Further, there is a need to determine the factors that maintain and exacerbate insomnia. The most important of these factors include sleep effort and sleep hygiene. Sleep effort is sleep-related performance anxiety, and it is defined as making an intense effort to sleep. It has two important elements: cognitive (“I must sleep”) and behavioral (performance effort) (22). In a study conducted on university students in Turkey, it was found that sleep effort showed a high positive correlation (*r* = 0.66) with insomnia severity (23). Additionally, some practices such as regular exercise and avoiding caffeine near bedtime make it easier to fall asleep and improve sleep quality (24). Such practices are collectively termed as sleep hygiene (25). Studies conducted on university students have determined that poor sleep hygiene is associated with poor sleep quality (23, 25). Additionally, in a study conducted on university students in Turkey, a significant positive correlation was observed (*r* = 0.47) between insomnia severity and poor sleep hygiene (25).

Type D personality is associated with negative health outcomes such as exhibiting negative health behaviors (e.g., taking up smoking or smoking more than usual), avoiding facilitating health behaviors (e.g., physical activity), and poorer coping with stress (26, 27). Individuals with Type D personality traits also use more passive or maladaptive avoidance coping strategies when faced with stress (27, 28). They may exhibit greater sleep effort and/or poor sleep hygiene, possibly in response to stress-related insomnia. This can lead to further exacerbation of insomnia. These individuals perceive stressful events as a higher level of threat or feel a higher level of stress as compared to others (29). There is some evidence that they have greater stress reactivity when faced with acute laboratory stress (30). Therefore, people with a higher number of Type D personality traits experience more negative emotions; they may be more affected by stressful events, and consequently develop an insomnia response. It is expected that Type D personality traits may be positively correlated with sleep reactivity.

Based on the above, the following four related concepts can be considered potential predictors of development of insomnia and severity of insomnia: (a) Type D personality, (b) sleep reactivity (vulnerability to stress-related insomnia), (c) sleep effort (sleep-related performance anxiety and intense effort to sleep) and, (d) sleep hygiene (adaptive behaviors for good sleep). However, to date, no study has yet examined the effect of these four factors on insomnia severity together. Consequently, in the present study, we aimed to evaluate the relationship of Type D personality, sleep reactivity, sleep effort, and sleep hygiene on insomnia severity in university students. This population represents a suitable sample to examine this association given that university students are at significant risk for developing insomnia due to varying school hours, active social lives, and demanding work schedules (31, 32).

We hypothesized that individuals with Type D personality traits have higher insomnia severity, sleep reactivity, sleep effort, and sleep hygiene scores. As the number of Type D personality traits increase, insomnia severity, sleep reactivity, sleep effort, and sleep hygiene scores increase. These four factors may predict the severity of insomnia. Sleep reactivity, sleep effort, and sleep hygiene may mediate the relationship between Type D personality traits and insomnia severity. These hypotheses are consistent with Spielman’s 3-P model. This model is a good framework for understanding the factors that initiate and maintain insomnia (8). This model states the following: predisposing factors such as family history and sleep reactivity (sensitivity) increase the risk of the onset of insomnia. Precipitating factors include medical, environmental, or psychosocial stress factors and personality traits that initiate insomnia. Perpetuating factors are some maladaptive behaviors (e.g., poor sleep hygiene and excessive sleep effort) that cause insomnia (8).

## Materials and methods

### Study design and participants

A cross-sectional online survey was conducted among university students between June 2022 and July 2022. The survey participants were students enrolled in various faculties of Gaziantep University, Turkey. The study was approved by Gaziantep University Clinical Research Ethics Committee (decision no: 2022/181, date: 25.05.2022). All participants were informed about the study before participating in the study. After completing the informed consent form prepared within the framework of the Helsinki Declaration, they participated in the study. A total of 474 students participated in the study.

All participants completed the sociodemographic data form, ISI, D Type Personality Scale (DS-14), FIRST, GSES, and SHI. Insomnia severity was calculated by ISI. The factors affecting insomnia severity were assessed by using DS-14, FIRST, GSES, and SHI. The complete sociodemographic and sleep characteristics of the study sample are presented in Table 1. The study participants (*n* = 474) had an average age of 21.22 years (standard deviation [SD] = 2.22, range = 18–37). Over 80% of the participants were female; of these, 97.68% were never married, and approximately 75% of the participants were living with family. Approximately 50% of the participants described themselves as a good sleeper, while about half said they had suffered from insomnia in the past month. The psychometric scale mean ± SD scores of the participants were 9.71 ± 5.15 for ISI, 24.59 ± 11.91 for DS-14, 22.52 ± 5.85 for FIRST, 4.45 ± 3.25 for GSES, and 20.55 ± 7.95 for SHI.

**Table 1:** Sociodemographic and clinical variables of all participants.

Variable	Mean ± SD
Age (years)	21.22 ± 2.22
BMI (kg/m^2^)	22.16 ± 3.80
**Sex**	***n* (%)**
Male	84 (17.72)
Female	390 (82.28)
**Marital status**
Married	11 (2.32)
Unmarried	463 (97.68)
**Living with**
Alone	50 (10.55)
With family	340 (71.73)
With friend	84 (17.72)
**Frequencies for chronic disease**
Yes	35 (7.38)
**Have you had insomnia in the last month?**
Yes	196 (41.35)
**Would you describe yourself as a good sleeper?**
Yes	218 (45.99)
**Are you currently taking any psychotropic drug?**
Yes	10 (2.11)
**Have you used any psychotropic drug for insomnia in the last month?**
Yes	12 (2.53)
**Have you ever used any psychotropic drug for insomnia in your life?**
Yes	15 (3.17)
**Primary measures**	**(Mean ± SD)**
ISI	9.71 ± 5.15
DS-14	24.59 ± 11.91
FIRST	22.52 ± 5.85
GSES	4.45 ± 3.25
SHI	20.55 ± 7.95

DS-14: D Type Personality Scale-14; ISI: Insomnia Severity Index; FIRST: Ford Insomnia Response to Stress Test; SHI: Sleep Hygiene Index; GSES: Glasgow Sleep Effort Scale.

## Measures

### Sociodemographic data form

A form requesting the sociodemographic information of the participants, including age, sex, and marital status was prepared by the researchers. Additionally, the participants answered the following questions about insomnia and their use of psychotropic drugs: (1) Have you suffered from insomnia in the past month? (2) In general, would you describe yourself as a good sleeper? (3) Have you used psychotropic medication for insomnia in the past month? (4) Have you ever used psychotropic medication for insomnia in your life? (5) Are you currently using any psychotropic medication for any other purpose?

### DS-14

Type D personality was assessed with the 14-item self-measurement tool DS-14 (9). This scale consists of two subscales, containing seven items each. The first subscale consists of negative affect (NA) items (“I often feel unhappy,” “I am often irritated,” “I take a gloomy view of things,” “I am often in a bad mood” etc.); the second subscale comprises social inhibition (SI) items (“I often talk to strangers,” “I often feel inhibited in social interactions,” “I find it hard to start a conversation,” “I am a closed kind of person,” etc.) (9). Two items in the SI subscale are reverse coded and a score between 0 and 28 is obtained from each subscale. Higher scores indicate more Type D personality traits. Participants who scored 10 or more on both subscales are categorized as individuals with Type D personality traits. The Cronbach’s α value of the Turkish validity and reliability DS-14 study was found to be = 0.82 for NA and 0.81 for SI, and test–retest results were r = 0.84/0.78 (33). In this study, we found excellent internal consistency for this scale (Cronbach’s α = 0.89, McDonald’s ω = 0.90).

### ISI

Insomnia symptoms were measured by using the ISI (34). This index consists of seven items and evaluates the severity of insomnia suffered by the respondent in the past 2 weeks. Each item is scored between 0 and 4 and the total score varies between 0 and 28. High scores indicate higher insomnia severity. Additionally, this scale includes four severity categories: absence of insomnia (0–7), subthreshold insomnia (8–14), clinical insomnia (15–21), and severe insomnia (22–28) (35). We found robust internal consistency for ISI in this study (Cronbach’s α = 0.83, McDonald’s ω = 0.84).

### FIRST

The FIRST is a self-report scale that assesses sleep reactivity (20, 36). It comprises nine items and measures the probability of developing insomnia before or after stressful events (e.g., “after a stressful experience during the day,” “before an important meeting the next day,” etc.). Each item is scored between 1–4 (1 = not likely, 2 = somewhat likely, 3 = moderately likely, and 4 = very likely), and the total score ranges from 0 to 36. High scores indicate high sleep reactivity or high insomnia susceptibility (19). We found excellent internal consistency for this scale in this study (Cronbach’s α = 0.87, McDonald’s ω = 0.88).

### SHI

The SHI is a 13-item self-report scale developed by Mastin et al. (37). It aims to question the respondent’s behaviors related to sleep hygiene. It has a 5-point Likert structure that measures how often the respondent performs these behaviors. The total score ranges from 13 to 65. Higher scores indicate poor sleep hygiene. Turkish validity and reliability study were performed by Özdemir et al. (25); they found the internal consistency coefficient in a community sample and patients with major depression to be 0.70 and 0.71, respectively (25). We found good internal consistency of this scale in this study (Cronbach’s α = 0.80, McDonald’s ω = 0.81).

### GSES

The GSES was developed by Bromfield and Espie to measure sleep effort (22). It consists of seven items and a 3-point Likert structure (0 = “not at all,” 1 = “to some extent,” and 2 = “very much”). The total score varies between 0 and 14 points, with higher scores representing higher sleep effort. Turkish validity and reliability of GSES was performed by Uygur et al. (21). In their study, the scale proved to have high internal consistency (Cronbach’s α = 0.82, McDonald’s ω = 0.83) (23). We found robust internal consistency of the GSES in this study (Cronbach’s α = 0.83, McDonald’s ω = 0.84).

### Statistical analysis

The collected data were analyzed by using SPSS 23.0 (IBM Co., Armonk, NY, United States). Descriptive statistical analysis was conducted to identify the characteristics of sociodemographic variables (sex, age, marital status, sleep situation). To determine whether parametric or nonparametric statistics should be used in further analysis, a normality test was performed. One-Sample Kolmogorov–Smirnov test showed that all the main variables had a normal distribution (*p* > 0.05). Consequently, *t*-tests were employed to examine the differences between individuals with Type D and non-Type D personality traits in terms of sleep effort, sleep hygiene, sleep reactivity, and insomnia severity. First, we divided all participants into those with (NA ≥10 and SI ≥10) and without Type D personality traits according to the DS-14 results and compared sociodemographic and sleep-related information between the groups. Additionally, we divided the participants into groups of those with and without insomnia according to ISI criteria (>14) and compared the information related to Type D personality traits and sleep in relation to these two groups. Pearson’s correlation was used to determine the relationship between the relevant variables. Subsequently, we conducted hierarchical multiple regression analysis to identify the associations between age, sex, sleep reactivity, Type D personality traits, sleep effort, sleep hygiene, and insomnia severity. It is widely recognized that sleep reactivity and Type D personality traits can increase the likelihood of developing insomnia, and factors such as poor sleep effort and hygiene can worsen the symptoms of insomnia. In the first stage, sex and age were entered into the model. In the second stage, sleep reactivity and Type D personality traits were added to the model. In the third stage, sleep hygiene and sleep effort were added to the model. We examined multicollinearity of covariates by using variance inflation factor (VIF) (38, 39). A *p* < 0.05 was considered statistically significant.

We used the SPSS PROCESS macro for mediation analyses (40). For conducting these analyses, indirect effects were measured by using 5,000 bootstrapped samples, model 4, and 95% confidence interval (CI) (41, 42). The hypothesized model examined whether sleep hygiene, sleep effort, and sleep reactivity mediated the relationship between Type D personality traits (DS-14 scores) and subjective insomnia severity (ISI score). Pathways were considered statistically significant if the 95% CI did not include zero.

## Results

### Comparison of sociodemographic and sleep information between the group with and without Type D personality

We did not observe significant differences between those with and without Type D personality regarding age, gender, body mass index, marital status, and having a chronic disease. ISI, Type D personality, sleep reactivity, sleep hygiene, and sleep effort scores were significantly higher in those with Type D personality than in those without Type D personality. Individuals with Type D personality traits defined themselves as poor sleepers to a large extent than those without Type D personality traits; a significant number of members of the first group also stated that they suffered from insomnia in the last month. Table 2 presents the details regarding the comparison of sociodemographic and sleep information between the group with and without Type D personality traits.

**Table 2:** Comparison of sociodemographic and sleep variables between the group with and without Type D personality.

Variables	Type D personality *N* = 234 *n* (%), Mean ± SD	Non-type-D personality *N* = 240 *n* (%), Mean ± SD	*p* value
Age	21.42 ± 5.56	21.37 ± 2.34	0.90
Female	200 (85.5%)	190 (79.2%)	0.07
BMI	21.91 ± 3.70	22.40 ± 3.87	0.16
Not married	228 (97.4%)	235 (97.9%)	0.72
Chronic disease	16 (6.8%)	19 (7.9%)	0.65
Suffered from insomnia last month	113 (57.7%)	83 (34.6%)	0.002
Good sleeper	94 (40.2%)	124 (51.7%)	0.01
Current psyc. treatment	7 (3%)	3 (1.2%)	0.18
Clinical insomnia	53 (22.6%)	26 (10.8%)	0.001
DS-14	34.20 ± 7.65	15.23 ± 6.72	<0.001
ISI	11.02 ± 5.05	8.42 ± 4.94	<0.001
FIRST	23.88 ± 5.73	21.20 ± 5.68	<0.001
SHI	22.89 ± 7.18	18.27 ± 8.02	< 0.001
GSES	5.32 ± 3.13	3.60 ± 3.14	<0.001

DS-14: D Type Personality Scale-14; ISI: Insomnia Severity Index; FIRST: Ford Insomnia Response to Stress Test; SHI: Sleep Hygiene Index; GSES: Glasgow Sleep Effort Scale.

### Correlation between instruments

In the correlation analysis, a positive correlation was found between all scales at the *p* < 0.05 level (see Table 3). The highest correlation between instruments was with sleep effort (*r* = 0.62) and the lowest correlation was with sleep hygiene (*r* = 0.33; Table 3).

**Table 3:** Correlations between instruments.

Instruments	Correlation coefficient (value of *p*)				
	DS-14	FIRST	GSES	SHI	ISI
DS-14	–				
FIRST	0.31 (<0.001)	–			
GSES	0.31 (<0.001)	0.27 (<0.001)	–		
SHI	0.26 (<0.001)	0.14 (0.002)	0.35 (<0.001)	–	
ISI	0.34 (<0.001)	0.39 (<0.001)	0.62 (<0.001)	0.33 (<0.001)	–

DS-14: D Type Personality Scale-14; ISI: Insomnia Severity Index; FIRST: Ford Insomnia Response to Stress Test; SHI: Sleep Hygiene Index; GSES: Glasgow Sleep Effort Scale.

### Hierarchical multiple regression

Hierarchical multiple regression analysis was conducted to examine the independent influence of age, sex, sleep reactivity, Type D personality, sleep effort, and sleep hygiene on insomnia severity. Most studies consider a VIF >10 as an indicator of multicollinearity; however, some choose a more conservative threshold of 5 or even 2.5. As a result of the analysis, the VIF of the final model was 1.2, which did not exceed 2.5, confirming that the problem of multicollinearity did not occur in this study (39).

In the first stage, age and sex were included in the model, and sex was found to be a positive predictor of insomnia severity (*β* = 0.15, *t* = 3.37, *p* = 0.001). The model explained 2% of the variance in insomnia severity. In the second stage, sleep reactivity (FIRST) and Type D Personality (DS-14) variables were added in the model. The predictive effect of sex on insomnia severity disappeared (*β* = 0.03, *t* = 0.69, *p* = 0.48). All variables (age, sex, sleep reactivity, and Type D personality traits) explained 20% of the variance in insomnia severity. When the possible effects of age and sex were controlled, these variables (sleep reactivity and Type D personality traits) explained 18% of the variance in insomnia severity (*R*^2^ = 0.20, *R*^2^ change = 0.18, *F*(4,474) = 29.91, *p* < 0.001). Finally, in the third stage, sleep effort and sleep hygiene were added to the model. All variables (age, sex, sleep reactivity, Type D personality traits, sleep effort, and sleep hygiene) explained 45% of the variance in insomnia severity. When age, sex, insomnia response to stress, and Type D personality traits were controlled, these variables (sleep effort and sleep hygiene) significantly explained 25% of the variance in insomnia severity (*R*^2^ = 0.45, *R*^2^ change = 0.25, *F*(6.474) =65.58, *p* < 0.001).

Sleep effort was the strongest predictor with the highest standardized coefficient of insomnia severity (*β* = 0.50, partial correlation = 0.44, partial correlation^2^ = 0.19). Sleep effort alone explained 19% of the variation in insomnia severity. Sleep reactivity is another important variable that independently predicted the severity of insomnia (*β* = 0.19, partial correlation = 0.17, partial correlation^2^ = 0.02). Sleep reactivity explained 2% of the variance in insomnia severity. Table 4 provides details of the hierarchical multiple regression analysis.

**Table 4:** Hierarchical regression analysis of insomnia severity (*N* = 474).

Predictors	Part-cor	*t*	*p*	β (std.)	*R*	*R* ^2^	*R*^2^ change	*F*
Step 1			0.003		0.15	0.02	0.02	5.72
Age	0.00	0.00	0.997	0				
Sex	0.15	3.37	0.001	0.15				
Step 2			0.000		0.45	0.20	0.18	29.91
Age	0.02	0.60	0.543	0.02				
Sex	0.02	0.69	0.485	0.03				
FIRST	0.28	6.96	0.000	0.30				
DS-14	0.22	5.33	0.000	0.23				
Step 3			0.000		0.67	0.45	0.25	65.58
Age	−0.01	−0.30	0.759	−0.01				
Sex	0.06	1.92	0.055	0.06				
FIRST	0.17	5.25	0.000	0.19				
DS-14	0.06	2.03	0.042	0.07				
GSES	0.44	13.12	0.000	0.50				
SHI	0.09	2.89	0.004	0.10				

DS-14:D Type Personality Scale-14; ISI: Insomnia Severity Index; FIRST: Ford Insomnia Response to Stress Test; SHI: Sleep Hygiene Index; GSES: Glasgow Sleep Effort Scale.

### Hypothesized model results (Type D personality>sleep effort>insomnia severity)

We observed a significant association between Type D personality traits and ISI (c path or total effect = 0.14, SE = 0.01, *p* < 0.001, 95% CI = 0.10, 0.18). Further, we observed a positive association between Type D personality traits and sleep effort (a-path, higher Type D personality traits associated with excessive sleep effort), as well as between sleep effort and ISI (b-path, with excessive sleep effort associated with more severe insomnia, see Table 5). The indirect effect (ab) of sleep effort on ISI was also significant (see Table 5); the direct effect including the mediator was reduced but still significant (c’ = 0.06, SE = 0.01, *p* < 0.001, 95% CI = 0.03, 0.10), indicating that the relationship between Type D personality traits and ISI was partially mediated by sleep effort.

**Table 5:** Statistics for hypothesized mediation models.

	Coeff	SE	Lower CI	Upper CI	Model summary
**D-type > Sleep effort > ISI**					
D-Type > sleep effort (a)	0.084	0.012	0.061	0.108	*R*^2^ = 0.095, *F*_(1, 472)_ = 49.41, *p* < 0.001
Sleep effort > ISI (b)	0.900	0.059	0.784	1.017	*R*^2^ = 0.405, *F*_(2, 471)_ = 159.99, *p* < 0.001
D-Type > ISI (c’)	0.070	0.016	0.038	0.102	
Indirect effect	0.076	0.013	0.050	0.102	
**D-type > Sleep hygiene > ISI**					
D-Type > sleep hygiene (a)	0.174	0.030	0.116	0.234	*R*^2^ = 0.068, *F*_(1, 472)_ = 34.46, *p* < 0.001
Sleep hygiene > ISI (b)	0.166	0.028	0.110	0.221	*R*^2^ = 0.174, *F*_(2, 471)_ = 49.44, *p* < 0.001
D-Type > ISI (c’)	0.117	0.019	0.080	0.154	
Indirect effect	0.029	0.009	0.014	0.047	
**D-type > Sleep reactivity > ISI**					
D-Type > sleep reactivity (a)	0.155	0.022	0.112	0.197	*R*^2^ = 0.099, *F*_(1, 472)_ = 51.77, *p* < 0.001
Sleep reactivity > ISI (b)	0.277	0.038	0.202	0.352	*R*^2^ = 0.202, *F*_(2, 471)_ = 59.56, *p* < 0.001
D-Type > ISI (c’)	0.103	0.019	0.066	0.140	
Indirect effect	0.043	0.009	0.026	0.061	
**Total effect**					
D-type > ISI (c)	0.146	0.019	0.109	0.182	*R*^2^ = 0.113, *F*_(1, 471)_ = 60.04, *p* < 0.001

DS-14: D Type Personality Scale-14; ISI: Insomnia Severity Index; LLCI: lower limit confidence interval; ULCI: upper limit confidence interval.

### Hypothesized model results (Type D personality>sleep hygiene>insomnia severity)

We observed a significant association between Type D personality traits and ISI (c path or total effect = 0.14, SE = 0.01, *p* < 0.001, 95% CI = 0.10, 0.18). We observed a positive association between Type D personality traits and sleep hygiene (a-path, higher Type D personality traits associated with bad sleep hygiene), as well as between sleep hygiene and ISI (b-path, with excessive sleep hygiene associated with more severe insomnia, see Table 5). The indirect effect (ab) of sleep hygiene on ISI was also significant (see Table 5); the direct effect including the mediator was reduced but still significant (c’ = 0.11, SE = 0.01, *p* < 0.001, 95% CI = 0.07, 0.15), indicating that the relationship between Type D personality traits and ISI was partially mediated by sleep hygiene.

### Hypothesized model results (Type D personality>sleep reactivity>insomnia severity)

We observed a significant association between Type D personality traits and ISI (c path or total effect = 0.14, SE = 0.01, *p* < 0.001, 95% CI = 0.10, 0.18). A positive association was observed between Type D personality traits and sleep reactivity (a-path, higher Type D personality traits associated with high sleep reactivity), as well as between sleep reactivity and ISI (b-path, high sleep reactivity associated with more severe insomnia, see Table 5). The indirect effect (ab) of sleep reactivity on ISI was also significant (see Table 5); the direct effect including the mediator was reduced but still significant (c’ = 0.10, SE = 0.01, *p* < 0.001, 95% CI = 0.06, 0.13), indicating that the relationship between Type D personality traits and ISI was partially mediated by sleep reactivity.

## Discussion

The study results indicate that individuals with Type D personality traits have greater sleep reactivity, poor sleep hygiene, and excessive sleep effort. At the same time, a higher number of Type D personality traits were associated with higher scores of sleep effort and sleep hygiene. To date, no study has focused on the mediating role of sleep reactivity, sleep hygiene, and sleep effort in association with Type D personality and insomnia. In this study, we found that sleep reactivity, sleep effort, and sleep hygiene partially mediated the relationship between Type D personality traits and insomnia severity.

### Type D personality and sleep reactivity

Type D personality traits, that include two constructs—NA and SI—may result in individuals possessing these traits to experience more stress. For example, in a study, it was determined that Type D personality is positively related to high stress levels at work (11). Exposure to stress can lead to serious somatic diseases such as ischemic heart disease and mental health problems such as depression, anxiety, and insomnia (43). Studies show that somatic diseases are more common in individuals with Type D personality (43). It has also been found that individuals with a Type D personality have a four times greater risk for insomnia. Insomnia is closely related to stress and can be induced and exacerbated by stress. Individuals with Type D personality traits may develop a more stress-related insomnia response because they feel more negative emotions toward events (44). It has been determined that there exists a difference in the regulation of the hypothalamic–pituitary–adrenal axis in individuals with Type D personality, and that NA and SI are associated with excessive cortisol secretion in stressful situations (30). Therefore, it can be expected that individuals with Type D personality are more likely to develop insomnia in stressful situations. In this study, we found sleep reactivity to be higher in participants with Type D personality, consistent with the above hypothesis. Additionally, this study is the first to show that an increased magnitude of Type D personality traits, causes sleep reactivity and insomnia severity increase.

### Type D personality, severity of insomnia, sleep effort, and sleep hygiene

One of the main findings of our study is that individuals with Type D personality suffer from more severe insomnia. Type D personality has previously been shown to be associated with insomnia. It has been determined that insomnia and Type D personality are associated with nurses and police officers (16). It has also been found that adolescents with Type D personalities have a higher risk for insomnia (17). The absence of age and sex differences in our comparison between individuals with and without Type D personality provided us an important opportunity to compare sleep parameters regardless of age and female sex since old age and the female sex are shown to be risk factors for poor sleep (45). Individuals with Type D personality may evaluate their health status and the course of their diseases worse as compared to those without Type D personality (46). Therefore, individuals with these personality traits may evaluate their insomnia symptoms as worse than actual after onset; subsequently, they may engage in a higher number of sleep effort behaviors as compared to the general population. It has been shown that increased sleep effort makes insomnia worse. Some maladaptive behaviors may lead to the continuation and exacerbation of insomnia after onset (25). For example, not complying with sleep hygiene rules such as not drinking caffeine close to bedtime, and not waiting for a long time to fall asleep are the most important maladaptive behaviors for insomnia (25). Moreover, individuals with Type D personality have lesser coping skills with problems and exhibit reduced healthy lifestyle behaviors, parallel to individuals with neuroticism (26, 27). Further, these individuals are more prone to unhealthy risky behaviors (43). Therefore, they may not follow sleep hygiene rules, consequently exacerbating insomnia.

One of the strengths of this study is that we examined the factors affecting insomnia by conducting hierarchical regression analysis. In this analysis, age did not affect sleep, while the female sex had a predictive effect on sleep. Previous studies showed that the most important predictors of insomnia are old age and the female sex. Sleep quality deteriorates and insomnia is more common in old age (47). Many studies have consistently found that females experience more insomnia than men, and that the female sex is a predictor of insomnia (47). Finding the female sex to be a predictor for insomnia in this study was consistent with evidence in the existing literature. However, when we included Type D personality traits and sleep reactivity in the model, the effect of the female sex on sleep disappeared. This finding showed us that other gender-neutral predictors can trigger insomnia. In the hierarchical regression analysis, it was sufficient to explain the severity of insomnia together with sleep reactivity at a rate of 45%. The fact that Type D personality, which has traits parallel to neuroticism, is a predictor for insomnia, was consistent with the evidence in the existing literature. Insomnia is a risk factor for depression and even suicide (25). Further, it negatively affects the course of the disease in somatic diseases. As individuals with Type D personality are prone to somatic diseases, suffering from insomnia may affect their illness and quality of life more negatively (44).

### Study limitations and future recommendations

This study has some limitations. First, as the data were collected *via* the online survey method, there are concerns regarding data quality. However, it is cheaper in terms of cost, and most importantly, it mediates the rapid contribution of research to the literature. Moreover, it has been proven in studies that online and traditional data collection show similar psychometric properties. Additionally, our implementations of several recommended procedures have been further implemented (prescreening questions alleviate these concerns). Third, given the relatively small sample size of this study, the study findings should be examined in larger samples. Fourth, cross-sectional analyses preclude the formation of conclusions regarding temporal precedence of personality, sleep effort, sleep hygiene, and sleep reactivity; therefore, longitudinal studies are needed to provide further support for the mediation models. Given that our study results only showed partial mediation of sleep effort, sleep hygiene, and sleep reactivity on the relationship between Type D personality traits and insomnia severity, future studies should examine other potential mediators (e.g., state–trait anxiety and depression severity, since depression and anxiety are more common in individuals with Type D personality (48)), that influence this pathway (49). Fifth, most of the participants in our study were female; it is noteworthy that in many studies on university students, the participants were female. This can be explained by the high rate of female participation in the study or the higher number of female university students. Sixth, the study results are not generalizable since the study participants were university students. Finally, objective measurements of sleep, such as polysomnography or actigraphy, were not performed to assess insomnia severity. Therefore, future studies should assess mediational models examined in this study by using objective measures of sleep (e.g., polysomnography).

## Conclusion

The present study demonstrated that individuals with Type D personality experience more severe insomnia, and that sleep reactivity, sleep effort, and sleep hygiene possibly mediates their association. Highly negative emotions as well as social inhibition may cause the individual with Type D personality to be strongly affected by events. Moreover, lower social support does not allow them to cope well with stress. This results in an increased likelihood of them developing insomnia. Although, cognitive behavioral therapy might help them to cope with stress and sleep reactivity, the tendency of a Type D personality to avoid healthy living habits, such as sleep effort and hygiene can exacerbate their insomnia. Therefore, informing these individuals about sleep hygiene and sleep effort could possibly alleviate their insomnia severity.

## Data availability statement

The original contributions presented in the study are included in the article/supplementary materials, further inquiries can be directed to the corresponding authors.

## Ethics statement

The study was approved by Gaziantep University Clinical Research Ethics Committee (decision no: 2022/181, date: 25.05.2022). The patients/participants provided their written informed consent to participate in this study.

## Author contributions

OU, HU, AB, and OH: conceptualization. OA: formal analysis. OU: investigation. OU, HU, and OH: methodology. OA, SC, and CD: supervision. OU, AB, and CD: writing – original draft. OU, OH, HU, and CD: writing – review and editing. All authors contributed to the article and approved the submitted version.

## Conflict of interest

The authors declare that the research was conducted in the absence of any commercial or financial relationships that could be construed as a potential conflict of interest.

## Publisher’s note

All claims expressed in this article are solely those of the authors and do not necessarily represent those of their affiliated organizations, or those of the publisher, the editors and the reviewers. Any product that may be evaluated in this article, or claim that may be made by its manufacturer, is not guaranteed or endorsed by the publisher.
